# Clinical markers of immunotherapy outcomes in advanced sarcoma

**DOI:** 10.1186/s12885-023-10758-w

**Published:** 2023-04-07

**Authors:** Marium Husain, Dionisia Quiroga, Han Gil Kim, Scott Lenobel, Menglin Xu, Hans Iwenofu, James L. Chen, Claire Verschraegen, David Liebner, Gabriel Tinoco

**Affiliations:** 1 Division of Medical Oncology, Department of Internal Medicine, The Ohio State University Comprehensive Cancer Center, 1800 Cannon Drive, Suite 1240C, 43210 Columbus, OH USA; 2 Department of Radiology, The Ohio State University Comprehensive Cancer Center, Columbus, OH USA; 3 Department of Pathology, The Ohio State University Comprehensive Cancer Center, Columbus, OH USA

**Keywords:** Soft tissue sarcomas, Immunotherapy, Biomarkers

## Abstract

**Background:**

Despite immunotherapy’s promise in oncology, its use for sarcoma remains challenging. There are no sarcoma-specific biomarkers for immune checkpoint inhibitors (ICI). Previously, we reported our institutional experience highlighting ICI activity in 29 patients with sarcoma. In this study, we explore responses to ICI based on ICI regimen and other covariates to identify significant clinical factors in advanced sarcoma outcomes.

**Methods:**

Patients in The Ohio State University Sarcoma Clinics were enrolled in the Sarcoma Retrospective ICI database from January 1, 2015 through November 1, 2021. Data included treatment regimen (single-agent ICI or ICI + combination) along with clinical covariates. ICI + combination was further categorized into ICI + medication, ICI + radiation, ICI + surgery, or ICI + multiple (more than 2 modalities). Statistical analysis included log-rank tests and proportional hazard regression. The primary objective was to evaluate overall survival (OS) and progression-free survival (PFS).

**Results:**

Of the patients in the database, 135 met inclusion criteria. We demonstrated improved OS in patients treated with ICI + combination (p = 0.014, median 64 weeks), but no effect on PFS (p = 0.471, median 31 weeks). Patients with a documented immune-related adverse event (irAE) of dermatitis had improved OS, but only in the ICI + combination cohort (p = 0.021). Patients who received single-agent ICI and whose change in the neutrophil-to-lymphocyte ratio (NLR) was less than 5 had an improved OS (p = 0.002); this was not seen in patients who received ICI + combination therapy (p = 0.441). There were no differences in OS based on age, gender, histology, or subcategories of ICI + combination. This was not the case for PFS; patients who received any ICI regimen and were younger than 70 had a worse PFS (p = 0.036) compared with their older counterparts in this dataset. Patients who developed an irAE, specifically colitis (p = 0.009), hepatitis (p = 0.048), or dermatitis (p = 0.003), had an improved PFS. There were no differences in PFS based on ICI regimen (or subcategories of ICI + combination), gender, histology, change in NLR, or grade of irAE.

**Conclusions:**

This retrospective study demonstrates that ICI + combination therapy can improve OS in some patients with advanced sarcoma. This is consistent with our prior results of ICI in sarcoma.

**Supplementary Information:**

The online version contains supplementary material available at 10.1186/s12885-023-10758-w.

## Background

Soft-tissue sarcomas (STSs) encompass a group of rare and heterogeneous malignant tumors that arise from the oncogenic transformation of mesenchymal tissue. These cancers account for less than 1% of all adult malignancies in the United States, with an estimated incidence of 13,130 new cases per year and approximately 5,350 deaths [1]. Over 100 distinct STS histological and molecular subtypes exist, each with unique clinical, therapeutic, and prognostic features [2]. Patients who develop metastatic disease have limited therapeutic options and median overall survival (OS) of about 18 months [1, 3–6]. Standard-of-care systemic chemotherapy agents include anthracyclines, ifosfamide, gemcitabine, taxanes, trabectedin, and pazopanib. The response rate remains low, and toxicities limit treatment dosing and length of therapy. This retrospective analysis will address the challenges and new development of immunotherapy for the treatment of sarcoma.

Two landmark trials (SARC028 and Alliance A091401) have demonstrated tumor response to immune checkpoint inhibitors (ICIs) as single-agent and combination regimens in certain sarcoma subtypes. However, there are no sarcoma-specific biomarkers for ICIs and programmed death-ligand 1 (PD-L1) expression did not correlate with clinical response [15, 16]. Indeed, a pooled analysis of clinical trials in STS demonstrated an inconsistent response to ICI based on the PD-L1 expression rate. Previously, we reported our institutional experience highlighting ICI activity in 29 patients with sarcoma [18]. We demonstrated a positive correlation between programmed death-1 (PD-1) expression and OS and progression-free survival (PFS) in sarcoma treated with PD-1 inhibitors, but not with PD-L1 expression as observed in other tumor types. Two other analyses of various sarcoma tissue samples showed a positive correlation between sarcomas that express PD-1/PD-L1 and T cell infiltration and activation [19, 20]. Patients with sarcomas containing increased copy numbers of the PD-1 gene have poorer survival outcomes [21]. Despite these observations, there are no reliable markers of response to immunotherapy in patients with sarcoma. In this updated study, we further explore OS and PFS outcomes of ICI-based therapy, focusing on the specific ICI regimen and clinical covariates to identify the significant clinical factors that could enlighten advanced sarcoma outcomes with immunotherapy.

## Methods

Patients seen at The Ohio State University Comprehensive Cancer Center Sarcoma Clinics were enrolled in the Sarcoma Retrospective ICI database from January 1, 2015, through November 1, 2021. Patients were included if they had received any ICI therapy. Therapy was categorized as single-agent ICI or ICI + combination. ICI + combination was further categorized into ICI + medication, ICI + radiation, ICI + surgery, or ICI + multiple (more than 2 modalities). Primary outcomes were OS and PFS. Clinical covariates assessed via the medical record were stage, age, gender, histology, change in the neutrophil-to-lymphocyte ratio (NLR), number of cycles, and immune-related adverse events (irAEs). NLR was calculated as the change in the neutrophil-to-lymphocyte ratio between day 0 and day 14 after ICI treatment administration. A cutoff ratio of 5 was used based on prior studies [11], with < 5% known to be associated with ICI response. There were 9 irAE types assessed: pneumonitis, colitis/diarrhea, abnormal thyroid function, hepatitis, dermatitis/rash, myalgia/arthralgia, neurological abnormalities, hypophysitis, and others.

### Statistical analysis

The primary objective was to evaluate overall survival and progression-free survival. We used log-rank tests and proportional hazard regression.

## Results

### Demographics

We screened 138 patients, and 135 met inclusion criteria (Table 1).

**Table 1:** Patient Characteristics

Patient Characteristics, *n*	
Number of patients screened	138
Number of patients deemed eligible	135
Sex (Female/Male) (%)	47/53
Median age at ICI start (yrs)	62
Stage 4	130
**Line of Systemic Therapy**, *n* (%)	
First	14 (10)
Second	36 (27)
Third line or beyond	85 (63)
**Histologies** (%)	
Undifferentiated pleomorphic sarcoma (UPS)	21 (19)
Leiomyosarcoma (LMS)	7 (7)
Liposarcoma (LPS)	11 (10)
Other	69 (64)
**ICI regimen**, *n* (%)	
Single-agent ICI	71 (53)
ICI + combination	64 (47)
**ICI + combination Breakdown**, *n* (%)	
ICI + medication	31 (59)
ICI + radiation	7 (15)
ICI + surgery	9 (13)
ICI + multiple	14 (13)
**Number of Cycles and NLR**, *n* (%)	
Received 3 or more cycles	76 (60)
NLR < 5 (%)	112 (92)
NLR ≥ 5 (%)	10 (8)

There were more male patients (53%) than females (47%). Most patients were younger than 70 years of age (71%). Most patients were metastatic/stage 4 (96%). Histology was categorized into undifferentiated pleomorphic sarcoma (UPS; 19%), leiomyosarcoma (LMS; 7%), liposarcoma (LPS; 10%), and other (68%). There were various other histologies, but each type had less than 6 patients; therefore, they were aggregated into the “other” category to facilitate statistical analysis (Table 2). The other histologies consisted of: angiosarcoma, chondrosarcoma, GIST, bone (osteosarcoma, Ewing), synovial, adenosarcoma, alveolar soft part sarcoma (ASPS), chordoma, clear cell, dendritic cell, epithelioid sarcoma, inflammatory myofibroblastic tumor, myxofibrosarcoma, PEComa, solitary fibrous tumor, and sarcoma not otherwise specified (NOS). 63% of patients were receiving ICI in the third-line setting or beyond, with only 10% receiving it as first-line therapy. 53% received single-agent ICI, and 47% received an ICI combined with another therapy (ICI + combination). ICI used were pembrolizumab, nivolumab, ipilumumab or combination ipilimumab and nivolumab. Of the patients that received ICI + combination, 59% received ICI + medications (either intravenous chemotherapy or targeted therapies: tyrosine kinase inhibitors, CDK4/6 inhibitors, mTOR inhibitors, estrogen receptor inhibitors, proteasome inhibitors, used either concurrently or sequentially), 15% received ICI + radiation (either concurrently or sequentially), 13% received ICI + surgery (either neoadjuvant or adjuvant), and 13% received ICI + multiple (a combination of 2 or more modalities). Radiation therapy and surgery were palliative in nature due to most patients having advanced stage disease at time of immunotherapy treatment. Most patients received 3 or more cycles of ICI therapy (any regimen; 56%). The NLR was less than 5 in 88% of patients and 12% had an NLR ≥ 5. The incidence of irAE was as expected for the ICI administered.

**Table 2:** Histology

Histology	Subtype	*n*
UPS		21
Leiomyosarcoma		7
	Non-uterine	4
	Uterine	3
Liposarcoma		11
	Dedifferentiated (DD) LPS	7
	Myxoid liposarcoma	2
	Well-differentiated (WD) LPS	1
	Inflammatory	1
Other		69
Angiosarcoma		12
	Cutaneous	7
	Radiation-induced	1
	Visceral	2
	Breast	2
Chondrosarcoma		7
	Conventional	3
	Dedifferentiated chondrosarcoma	3
	Mesenchymal chondrosarcoma	1
GIST		5
Bone		5
	Osteosarcoma	4
	Ewing	1
Rhabdomyosarcoma		0
Synovial		4
	Biphasic	1
	Monophasic	1
	Synovial, NOS	2
Other		36
	Adenosarcoma	2
	Alveolar soft part sarcoma	2
	Chordoma	3
	Clear cell sarcoma	1
	Dendritic cell sarcoma	1
	Epithelioid sarcoma	3
	Inflammatory myofibroblastic tumor	1
	Myxofibrosarcoma	6
	PEComa	2
	Solitary fibrous tumor	1
	Sarcoma, NOS	14

### Overall survival

Patients treated with ICI + combination had an improved OS (median 64 weeks) compared to single-agent ICI (median 37 weeks) (p = 0.014) (Table 3).

**Table 3:** Overall Survival (OS)

Overall Survival	
**Category**	**Median OS**, in weeks (range, in weeks)
Single-agent ICI	37 (33–41)
ICI + combination	64 (38–90)
3 or more cycles; any ICI regimen	74 (55–93)
Less than 3 cycles; any ICI regimen	12 (5–20)
NLR < 5	60 (41–79)
NLR ≥ 5	11 (0–34)

**Fig. 1:**
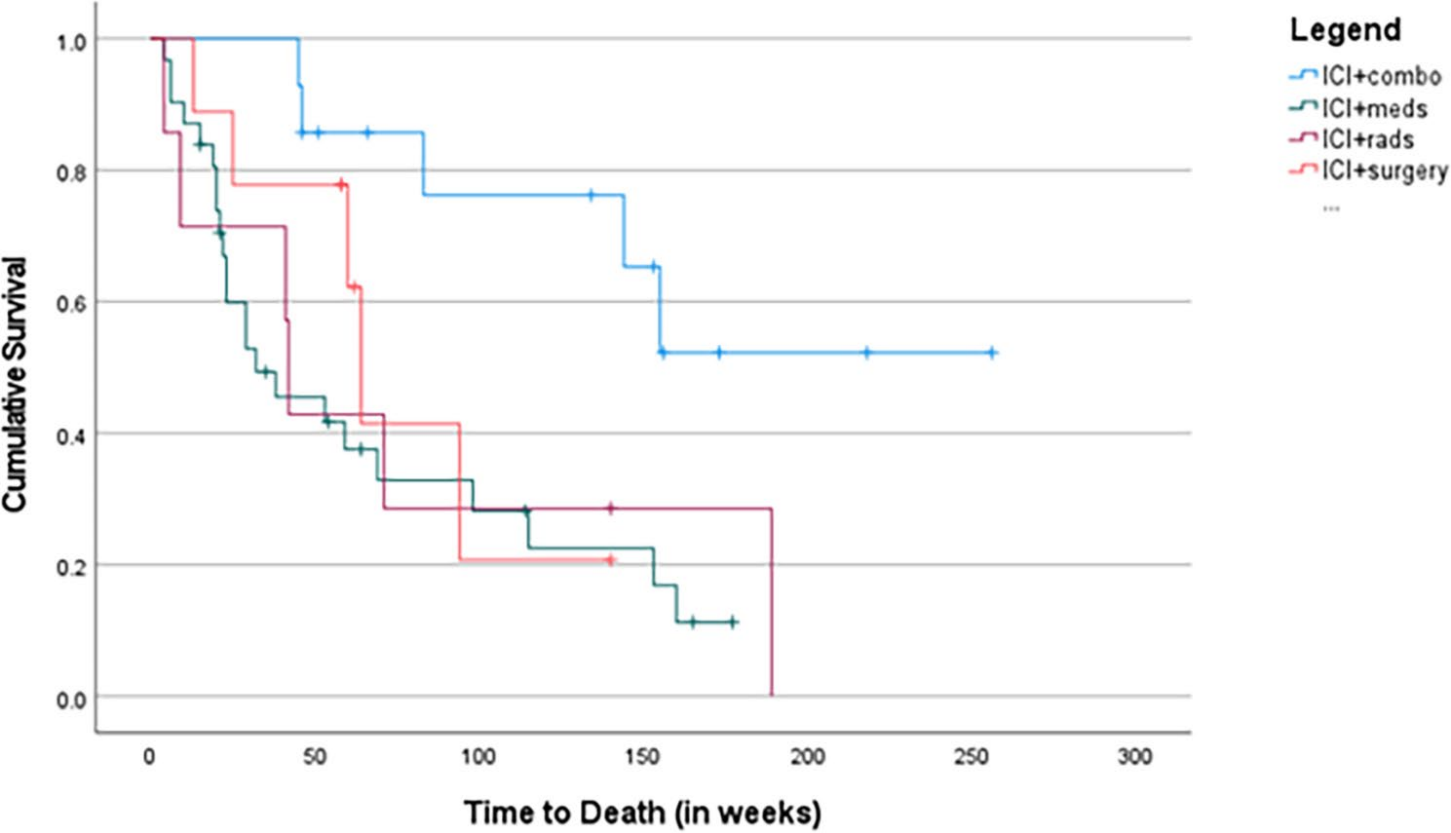
OS Across Various ICI Regimens

Specifically, patients who received ICI in combination with 2 or more modalities (ICI + multiple) had the best outcome (Fig. 1).

**Fig. 2:**
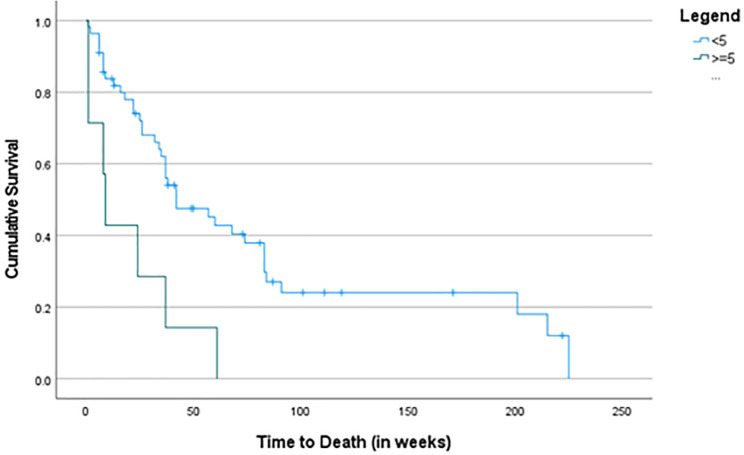
Association Between OS and NLR

Whether patients had received single-agent ICI or ICI + combination, those who received 3 or more cycles of ICI had an improved OS—a median of 74 weeks compared to 12 weeks for those patients who received less than 3 cycles (p < 0.001) (Table 2). Patients who received single-agent ICI and whose change in NLR was < 5 had an improved OS (median 42 weeks) compared to those whose NLR was ≥ 5 (median 9 weeks) (p = 0.002) (Fig. 2); however, this was not seen in patients who received ICI + combination therapy (p = 0.441).

**Fig. 3:**
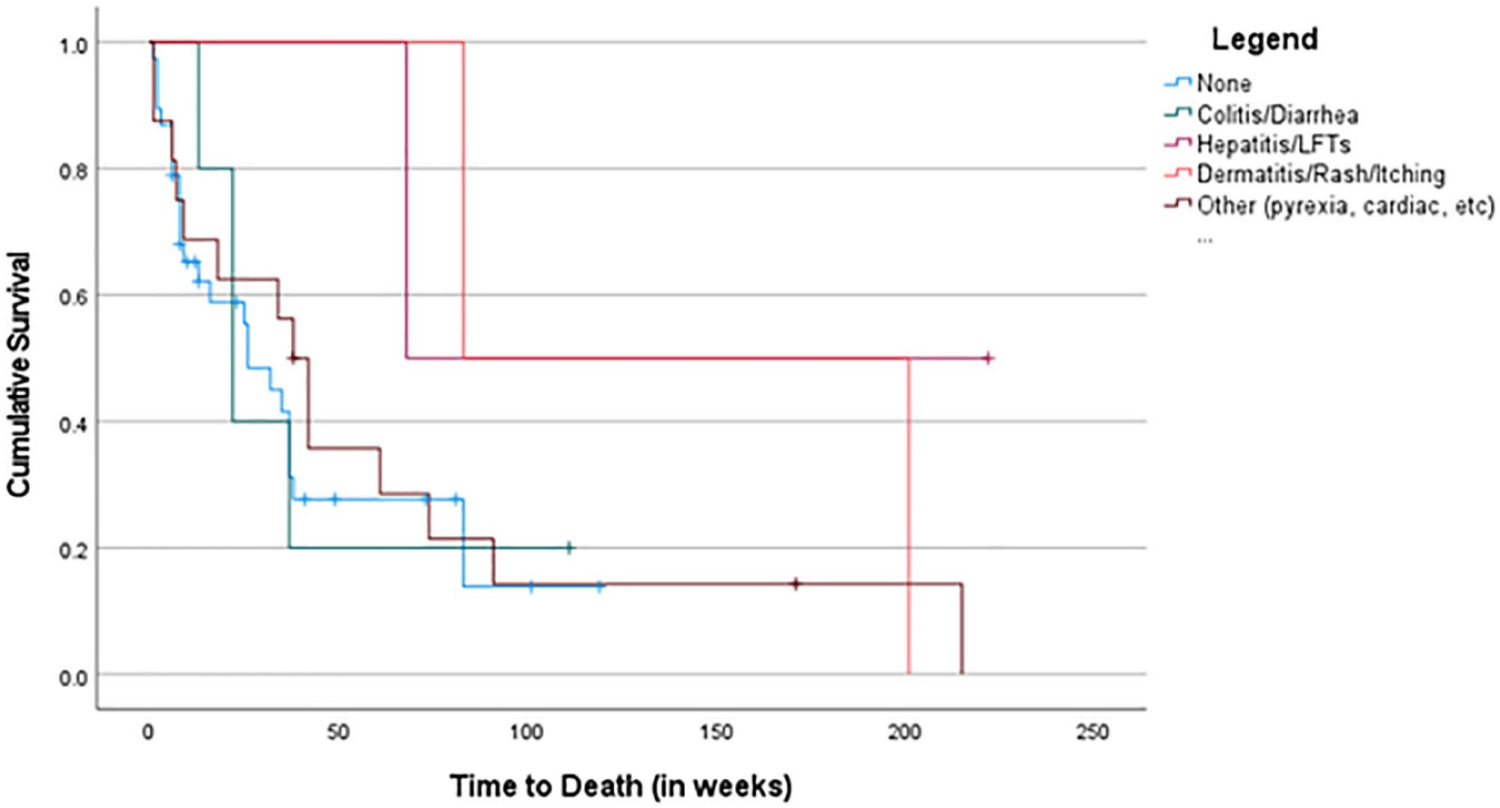
Survival Curves Based on irAE in ICI-Only Cohort

**Fig. 4:**
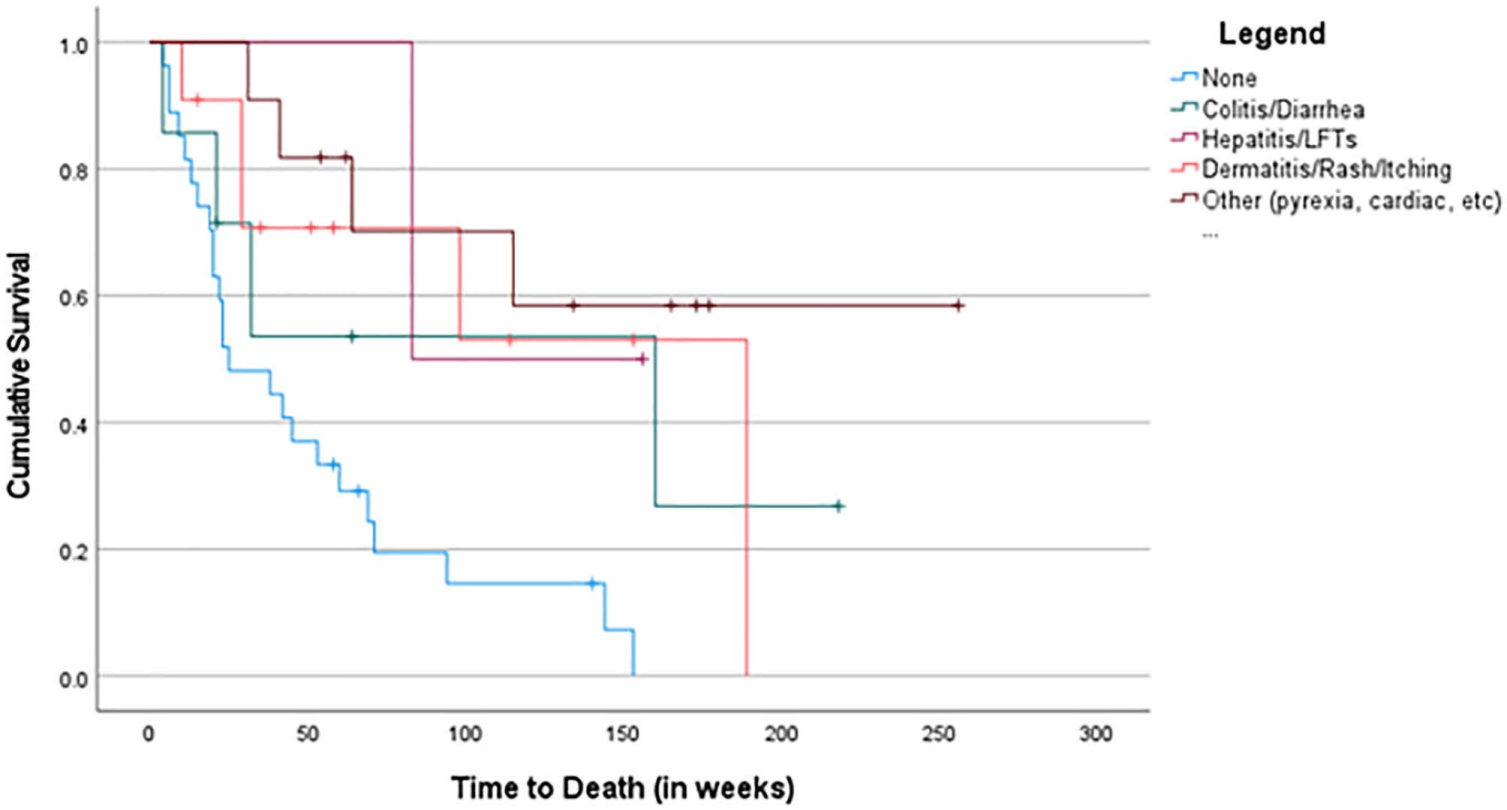
Survival Curves Based on irAE in ICI + Combination Cohort

Patients who had a documented irAE of colitis/diarrhea (p = 0.009), hepatitis (p = 0.042), or dermatitis/rash (p = 0.010) were associated with an improved OS across the whole cohort (Figs. 3 and 4).

More specifically, patients treated in the ICI + combination group and who developed dermatitis had an improved OS (p = 0.021) (Fig. 4).

There were no differences in OS based on age (p = 0.354), gender (p = 0.628), histology (p = 0.342), or subcategories of ICI + combination.

### Progression-free survival

In this analysis, we demonstrated an improvement in PFS in patients with UPS (median 68 weeks) compared to the other histologies (p = 0.017) (Table 4).

**Table 4:** Progression-Free Survival (PFS)

Progression-Free Survival	
**Category**	**Median PFS**, in weeks (range, in weeks)
Any ICI regimen for patients < 70yrs	16 (11–21)
Any ICI regimen for patients ≥ 70yrs	42 (4–80)
3 or more cycles; any ICI regimen	27 (0–54)
Less than 3 cycles; any ICI regimen	7 (5–9)

This statistically significant signal was demonstrated in patients who received single-agent ICI (p = 0.009) but not those who received ICI + combination (p = 0.698). Patients who received any ICI regimen and were younger than 70 had a worse PFS—a median of 16 weeks compared to 42 weeks in patients 70 years and older (p = 0.036). There were no differences in PFS based on gender (p = 0.489). There was no effect on PFS between single-agent ICI and ICI + combination (regardless of subcategory) (p = 0.296). Patients who received 3 or more cycles of ICI—whether single-agent ICI or ICI + combination–had an improved PFS, a median of 27 weeks compared to 7 weeks (p < 0.001). There was no significant association between PFS and NLR (p = 0.649).

Patients who developed an irAEs, specifically colitis (p = 0.009), hepatitis (p = 0.048), and dermatitis (p = 0.003), had an improved PFS across the whole cohort. There was a higher PFS seen in patients who received ICI + combination and experienced dermatitis (p = 0.007) and irAEs in the “other” category (p = 0.033) compared to the single-agent ICI regimen. The development of any irAEs was positively associated with the number of doses of ICI administered (≥ 3) (p = 0.003), as expected. However, there was no correlation with the type of ICI agent (p = 0.134).

## Discussion

We performed a retrospective study of patients with advanced sarcoma who received ICI therapy and evaluated OS/PFS outcomes based on the type of ICI regimen and clinical covariates. We demonstrated that patients who received ICI + combination therapy had an improved OS but saw no effect on PFS. Patients who received 2 or more therapy modalities with ICI (that is, chemotherapy/targeted therapy, radiation, or surgery) had improved outcomes compared to ICI alone or ICI with a single modality. Radiation therapy and surgery were palliative in nature and we demonstrated a potential overall survival benefit for patients with advanced sarcoma who received an ICI + combination therapy. This was also demonstrated in a systematic review where ICI, in combination with chemotherapy, had a significant overall response rate [12].

We demonstrated the importance of a low NLR, which is known to be associated with an improvement in OS, but only in patients who received single-agent ICI. This benefit was not demonstrated in patients who received ICI + combination, possibly because chemotherapy and radiation influence bone marrow function. Our data suggest that NLR can play a role as a prognostic biomarker in patients starting ICI treatment. This is consistent with prior studies that have shown an association between NLR and OS, where patients who had a moderate decrease in NLR during treatment with ICI were found to have the longest survival and patients with a significant decrease or increase in NLR had shorter OS [22]. NLR could be further evaluated as a biomarker of outcomes across a larger sample size of sarcoma populations receiving single-agent ICI therapy.

We found no statistically significant association between PFS and ICI regimen, except in patients with UPS. In this histologic group, we saw a longer median PFS compared to the other subtypes and our data is consistent with prior studies that patients with UPS have a positive response to ICI compared to other subtypes [7, 8, 13, 14]. Further, patients 70 years and older had a significantly improved PFS compared to younger patients. It is unclear at this time why older patients would have a better outcome. ICI therapy could be considered in elderly patients if indicated. However, this finding does not contradict current studies on PFS as a surrogate for OS. Our sample size is too limited to make conclusions on surrogate endpoints in soft tissue sarcoma.

NLR might not be helpful as a biomarker for PFS in sarcoma as there was no significant association. Further evaluation with a larger sample size is warranted given prior studies showing an association between NLR and PFS in non-small cell lung cancer, demonstrating that a lower ratio is associated with improved PFS [23].

We demonstrated an association between OS/PFS and the number of ICI cycles administered. However, this is a tautology and does not represent a novel finding in sarcoma management. We present the findings in the paper to highlight both the significant and clinically insignificant findings in this retrospective analysis.

The occurrence of certain irAEs were associated with improvements in OS and PFS, particularly dermatitis. Colitis and hepatitis were also associated with improved OS and PFS across the cohort. In those patients who received ICI + combination therapy, the “other” irAEs category was associated with an improved OS and PFS. As demonstrated in prior studies, the occurrence of certain irAEs is reflective of an immune system boost from ICI therapy and suggests increased activity against cancer. Our ICI cohort results are consistent with the historical data from our previous study [10]. It can also be suggested that patients who responded to ICI received more doses and therefore developed more irAEs.

There were limitations to this study. Our sample size of 135 patients is small; however, this was an improvement from our prior study looking at ICI outcomes in 26 patients with sarcoma. Some patients were censored during analyses due to limited clinical information. Due to the rarity of sarcoma, most subtypes were not included as separate histologies in these analyses as there were too few patients to reach statistically significant results. Therefore, less frequent histologies were categorized as the “other” group. Histologies that are associated with positive outcomes from ICI, like ASPS, were not adequately represented in this sample to demonstrate a meaningful outcome with ICI therapy. However, despite this arrangement, our data for PFS was consistent with prior studies demonstrating that certain subtypes, like UPS, have a more favorable response to ICI therapy compared to others, such as LMS.

## Conclusions

This retrospective study demonstrates that ICI + combination therapy can improve OS in sarcoma. Benefits in OS/PFS were seen in patients who developed certain irAEs. Those with decreased changes in NLR also had improved OS, demonstrating a potential prognostic biomarker. Further studies will focus on evaluating larger sample sizes to evaluate more subtypes of sarcoma and to better understand the role of NLR in prognostication in sarcoma.

## Electronic supplementary material

Below is the link to the electronic supplementary material.

Supplementary Material 1

## Data Availability

The datasets used and/or analyzed during the current study are available from the corresponding author on reasonable request.

## Abbreviations

ICI: Immune checkpoint inhibitor
irAE: Immune-related adverse event
LMS: Leiomyosarcoma
LPS: Liposarcoma
NLR: Neutrophil-to-lymphocyte ratio
OS: Overall survival
PD-1: Programmed death-1
PD-L1: Programmed death-ligand 1
PFS: Progression-free survival
STS: Soft-tissue sarcoma
UPS: Undifferentiated pleomorphic sarcoma
