# Supplementary material for: Clinical markers of immunotherapy outcomes in advanced sarcoma
Source: BMC Cancer. 2023 Apr 7;23:326. doi: 10.1186/s12885-023-10758-w (PMC10080913; doi:10.1186/s12885-023-10758-w)
Supplement: Supplementary file 1 — Supplementary Material 1 [file 12885_2023_10758_MOESM1_ESM.docx]

**Supplemental Table 1: Immune Checkpoint Inhibitor (ICI) Systemic Therapies**

| **Systemic Therapy Types** | **Doses** | **N** | | | **Average # Cycles** | | |
| --- | --- | --- | --- | --- | --- | --- | --- |
| **Single-agent ICI, N = 71** | | | | | | | |
| Pembrolizumab | 200mg IV q3 weeks | 33 | | | 6 | | |
| Nivolumab | 3 mg/kg IV q28 days | 26 | | | 4.9 | | |
| Nivolumab + Ipilumumab | Nivolumab 3mg/kg; Ipilumuab 1mg/kg IV q3 weeks | 8 | | | 2.375 | | |
| Pembrolizumab | 400mg IV q6 weeks | 4 | | | 1.5 | | |
|  | **ICI + medications N = 21** |  | | |  | | |
| Regorafenib + Nivolumab | Nivolumab 3kg/mg IV q28 days; Regorafenib 40mg PO daily (Days 1-21 of a 28-day cycle) | 1 | | | 10 | | |
| Nivolumab + Imatinib | Nivolumab 3mg/kg IV q28 days; Imatinib 400mg PO daily | 1 | | | 2 | | |
| Nivolumab + Crizotinib | Nivolumab 3mg/kg IV q28 days; Crizotinib 250mg PO daily | 1 | | | 3 | | |
| Nivolumab + Paclitaxel | Nivolumab 3mg/kg (Day 1); Paclitaxel 126mg (Days 1, 8, 15 of a 28-day cycle) | 1 | | | 3 | | |
| Nivolumab + Sunitinib | Nivolumab 3mg/kg IV q28 days; Sunitinib 25mg PO daily | 1 | | | 2 | | |
| Pembrolizumab + Eribulin | Pembrolizumab 200mg IV q3 weeks (Day 1); Eribulin 1.5mg/kg2 (Days 1 and 8 of 21-day cycle) | 1 | | | 2 | | |
| Pembrolizumab + Ixazomib | Pembrolizumab 200mg IV q3 weeks; Ixazomib 3mg (Days 1, 8, 15 of a 28-day cycle) | 1 | | | 2 | | |
| Pembrolizumab + Letrozole + Everolimus | Pembrolizumab 200mg IV q3 weeks; Letrozole 10mg; Everolimus 10mg | 1 | | | 2 | | |
| Pembrolizumab + Palbociclib | Pembrolizumab 200mg IV q3 weeks; Palbociclib 125mg (Days 1-21 of a 28-day cycle) | 4 | | | 11 | | |
| Pembrolizumab + Pazopanib | Pembrolizumab 200mg IV q3 weeks; Pazopanib 200mg PO daily | 2 | | | 12.5 | | |
| Pembrolizumab + Pazopanib | Pembrolizumab 200mg IV q3 weeks; Pazopanib 400mg PO daily | 5 | | | 4.6 | | |
| Pembrolizumab + Sunitinib | Pembrolizumab 200mg; Sunitinib 200mg PO daily | 1 | | | 3 | | |
| Pembrolizumab + Denosumab | Pembrolizumab 400mg IV q6 weeks; Denosumab 120mg | 1 | | | 2 | | |
| **ICI + Multiple N = 21** | | | | | | | |
| Pembrolizumab | 200mg IV q3 weeks | | 5 | | 7.4 | | |
| Pembrolizumab | 400mg IV q6 weeks | | 1 | | 12 | | |
| Nivolumab | 3 mg/kg IV q28 days | | 6 | | 8.33 | | |
| Nivolumab + Ipilumumab | Nivolumab 3mg/kg; Ipilumumab 1mg/kg IV q3 weeks | | 1 | | 2 | | |
| Pembrolizumab + Paclitaxel + Carboplatin | Pembrolizumab 200mg; Paclitaxel 150mg/m2; Carboplatin AUC 5; IV q3 weeks | | 1 | | 5 | | |
| Pembrolizumab + Pazopanib | Pembrolizumab 200mg IV q3 weeks; Pazopanib 200mg PO daily | | 1 | | 2 | | |
| Pembrolizumab + Pazopanib | Pembrolizumab 200mg IV q3 weeks; Pazopanib 400mg PO daily | | 3 | | 3.33 | | |
| Pembrolizumab + Pazopanib | Pembrolizumab 200mg IV q3 weeks; Pazopanib 600mg PO daily | | 1 | | 8 | | |
| Pembrolizumab + Pazopanib | Pembrolizumab 200mg IV q3 weeks; Pazopanib 800mg PO daily | | 1 | | 4 | | |
| Pembrolizumab + Pazopanib | Pembrolizumab 400mg IV q6 weeks; Pazopanib 800mg PO daily | | 1 | | 13 | | |
| **ICI + Surgery N = 11** | | | | | | | |
| Nivolumab + Ipilumumab | Nivolumab 3mg/kg; Ipilumumab 1mg/kg IV q3 weeks | | | 3 | 2.667 | | |
| Pembrolizumab | 200mg IV q3 weeks | | | 6 | 10.667 | | |
| Nivolumab | 3 mg/kg IV q28 days | | | 2 | 33 | | |
| **ICI + Radiation N = 5** | | | | | | |  |
| Nivolumab | 3mg/kg IV q28 days | | 1 | | | 10 |  |
| Pembrolizumab | 200mg IV q3 weeks | | 4 | | | 7.75 |  |

**Abbreviations:** IV, intravenous; q, every; PO, by mouth
